# Functional Magnetic Composites Based on Hexaferrites: Correlation of the Composition, Magnetic and High-Frequency Properties

**DOI:** 10.3390/nano9121720

**Published:** 2019-12-02

**Authors:** Lyudmila Yu. Matzui, Alex V. Trukhanov, Olena S. Yakovenko, Ludmila L. Vovchenko, Volodymyr V. Zagorodnii, Victor V. Oliynyk, Mykola O. Borovoy, Ekaterina L. Trukhanova, Ksenia A. Astapovich, Dmitry V. Karpinsky, Sergei V. Trukhanov

**Affiliations:** 1 Physics Department, Taras Shevchenko National University of Kyiv, Volodymyrska Str. 64/13, 01601 Kyiv, Ukraine; fix.tatyana@gmail.com (L.Y.M.); svtruhanov@yandex.ru (O.S.Y.); vovchenko@yandex.ru (L.L.V.); kastor1986@yandex.ru (V.V.Z.); njsya99@gmail.com (V.V.O.); truhanov86@gmail.com (M.O.B.); 2 Department of Technology of Electronics Materials, National University of Science and Technology “MISiS”, Leninskii av., Moscow 4119049, Russia; katu-shkak@mail.ru (E.L.T.); sv_truhanov@mail.ru (S.V.T.); 3 SSPA “Scientific and Practical Materials Research Centre of the NAS of Belarus”, P. Brovki Str. 19, 220072 Minsk, Belarus; ks.astapovich@gmail.com (K.A.A.); dmitry.karpinsky@gmail.com (D.V.K.); 4 Scientific and Educational Center "Nanotechnology", South Ural State University, Lenin av. 76, Chelyabinsk 454080, Russia; 5 Department of Functional Electronics Materials, National Research University of Electronic Technology “MIET”, Zelenograd 124498, Russia

**Keywords:** magnetic composites, polymer-matrix composites (PMCs), magnetic properties, high-frequency properties

## Abstract

The paper describes preparation features of functional composites based on ferrites, such as “Ba(Fe_1−x_Ga_x_)_12_O_19_/epoxy,” and the results of studying their systems; namely, the correlation between structure, magnetic properties and electromagnetic absorption characteristics. We demonstrated the strong mutual influence of the chemical compositions of magnetic fillers (Ba(Fe_1−x_Ga_x_)_12_O_19_ 0.01 < *x* < 0.1 solid solutions), and the main magnetic (coercivity, magnetization, anisotropy field and the first anisotropy constant) and microwave (resonant frequency and amplitude) characteristics of functional composites with 30 wt.% of hexaferrite. The paper presents a correlation between the chemical compositions of composites and amplitude–frequency characteristics. Increase of Ga-content from *x* = 0 to 0.1 in Ba(Fe_1−x_Ga_x_)_12_O_19_/epoxy composites leads to increase of the resonant frequency from 51 to 54 GHz and absorption amplitude from −1.5 to −10.5 dB/mm. The ability to control the electromagnetic properties in these types of composites opens great prospects for their practical applications due to high absorption efficiency, and lower cost in comparison with pure ceramics oxides.

## 1. Introduction

One of the major tasks in creating modern mobile communication devices is the development of new materials which can work in a wide (up to 100 GHz) frequency range in switches, circulators, phase shifters, transceivers, antennas and effective electromagnetic radiation (EMR) absorbers, which improve the electromagnetic compatibilities of devices [1,2,3,4,5]. M-type hexagonal ferrites are one of the most prospective electromagnetic materials for application in the centimeter and millimeter wave range due to the large values of permeability and magnetization, and good dielectric properties at microwave frequencies [6,7,8,9,10]. These materials are magnetically hard with high coercivity and magnetic permeability, and are also characterized by high values of magnetocrystalline anisotropy along the *c*-axis of the hexagonal structure. Barium hexaferrite BaFe_12_O_19_ (BaM) is the first compound of this type which has been studied in detail and is now widely used. It has a high value of saturation magnetization (Ms) of 72 emu/g (1 emu/g = 1 (A × m^2^)/kg) and a high Curie temperature of 450 °C. BaM shows large magnetocrystalline anisotropy, about 17 kOe along the *c*-axis [10]. Natural ferromagnetic resonance (NFMR) in M-type barium hexaferrite occurs in U-band (EHF range, 47–50 GHz) due to the anisotropy of the *c*-axis, and therefore, these materials can be used as microwave EMR absorbers [11,12,13]. Doping of barium hexaferrite with various diamagnetic ions (DI), such as Al^3+^, In^3+^, Ga^3+^, Co^2+^ and Sc^3+^ makes it possible to adapt the magnetic characteristics, and consequently, to control the range of operating frequencies up to 70 GHz [14,15,16,17,18,19,20]. As it was shown in [21], the transmission and absorption spectra of Ba(Fe_1−x_DI_x_)_12_O_19_ demonstrate, depending on the substitution type and ratio, a change in the frequency and maximum absorption (associated with the NFMR) owing to a corresponding change in magnetocrystalline anisotropy (MCA).

The inclusion of Ba(Fe_1−x_DI_x_)_12_O_19_ powders into the polymer matrix opens up new application possibilities of such composites [22,23,24,25,26,27]. The advantages of polymer-bonded composites with BaM are: their ability to be molded into complex shapes and sizes; low weights and relatively low prices; sharply reduced dielectric loss compared to bulk ferrites; and stable microwave absorption properties due to the domination of NFMR absorption in the loss mechanism of the absorbing ferrite materials. The excellent features of ferrite/polymer composites make them very attractive for use not only as magnetic materials but also as microwave absorbing materials. Many studies were carried out to investigate the effects of ferrite volume fraction in the composite on microwave absorption. The importance of such materials was confirmed by numerous extensive studies developing new effective microwave-absorbing ferrite/polymer composites [28,29,30]. 

Previously, we demonstrated results about the magnetic and microwave properties of the Ga-substituted hexaferrites as bulk (ceramics). That study focused on principally new objects—composites based on Ga hexaferrite powders dispersed in epoxy. We examined the electromagnetic properties and microwave absorbing characteristics in Ga-substituted barium hexaferrite with epoxy (Ba(Fe_1−x_Ga_x_)_12_O_19_/epoxy) composites in the frequency range up to 67 GHz. Moreover, the correlation between the concentration x of Ga diamagnetic ions (0 < *x* < 0.1) and the electromagnetic properties of the composites was established.

## 2. Materials and Methods 

The Ba(Fe_1−x_Ga_x_)_12_O_19_ 0 < *x* < 0.1 powders we investigated were obtained from high purity oxides Fe_2_O_3_, Ga_2_O_3_ and carbonate BaCO_3_ via two-step solid state reactions. The precursor manufacturer was Xiamen Ditai Chemicals Co., Ltd (Xiamen, China). The oxides and carbonate were mixed in the design ratio. Then, the annealing was performed for 6 h at 1200 °C in air. The final synthesis was carried out during 6 h at 1300 °C in air. The samples were slowly cooled after the synthesis (100 °C/h) [31].

Epoxy-based composites (CMs) containing the Ga-substituted hexaferrites in low-viscosity epoxy (L285, Lange+Ritter GmbH, Gerlingen, Germany) with an appropriate cross-linking agent (hardener) H285, were produced for this study. Namely, Ba(Fe_1−x_Ga_x_)_12_O_19_/epoxy CMs with 30 wt.% of Ba(Fe_1−x_Ga_x_)_12_O_19_ were obtained by mixing in solution with additional sonication. The manufacturing process was as follows. At first, an appropriate amount of L285 epoxy resin was pre-dissolved in acetone. Further, BaFe_12−x_Ga_x_O_19_ was introduced into solution and sonicated in a BAKU ultrasonic bath for 1 h with 40 kHz frequency and 50 W power. Finally, curing agent H285 was added (40% by weight of L285). To complete the polymerization, one day after the CMs were prepared, they were heated for 5 h at a temperature gradually increased from 40 to 80 °C.

The crystal structures of Ba(Fe_1−x_Ga_x_)_12_O_19_ filler powders and the samples of CMs were investigated by X-ray diffraction, which was carried out using a DRON-4-07 X-ray diffractometer (Bourevestnik, St. Petersburg, Russia) with Co K_α_ filtered radiation (λ = 1.7902 A) at room temperature.

The morphology of Ba(Fe_1−x_Ga_x_)_12_O_19_ powders and Ba(Fe_1−x_Ga_x_)_12_O_19_/epoxy composites was characterized by Mira 3 Tescan scanning electron microscope (SEM) (TESCAN ORSAY HOLDING, Brno–Kohoutovice, Czech Republic).

**Figure 1:**
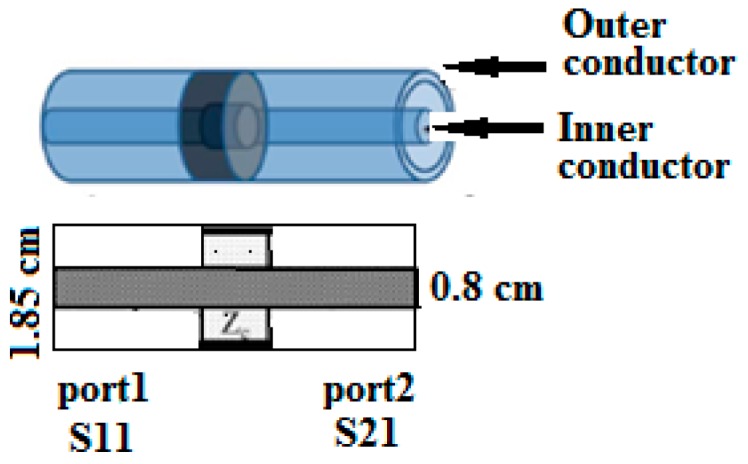
The scheme of measurements with the coaxial transmission line (the measuring cell and the sample are given schematically).

Keysight PNA N5227A vector network analyzer (Keysight Technologies, Inc. Santa Rosa, CA, USA) was used to determine the electromagnetic parameters of the composites in the frequency range of 1–67 GHz by the transmission line method. Full, two-port calibration was initially performed on the test setup to remove errors due to the directivity, source match, load match, isolation and frequency response of each of the forward and reverse measurements. The coaxial measuring cell (Figure 1) had the inner conductor diameter of 0.8 cm and the outer conductor diameter of 1.85 cm (thickness of the samples was fixed at 1 cm). The tested samples were shaped into a form of a hollow cylinder, which tightly fit into the coaxial measuring cell. The measured reflection coefficient (S_11_) and transmission coefficient (S_21_) of each composite was converted into material shielding efficiency (SE_T_), reflection coefficient (SE_R_) and coefficient of absorption (SE_A_) according to the equations:

SE_T_ = 20log(|S_21_|), (1)

SE_R_ = 10log(1 − |S_11_|), (2)

SE_A_ = −|SE_T_| − |SE_R_|. (3)

The magnetic properties (field dependencies of the magnetization) were determined using a universal cryogenic high-field measuring system (Liquid Helium Free High Field Measuring System (B14T) by Cryogenic Ltd., London, UK) at the temperature of 300 K in external magnetic fields up to 5 T (field magnetization curve). The size of each sample for magnetic measurements was 5.2 × 2.6 × 2.6 mm^3^.

## 3. Results and Discussion

### 3.1. Structural Characteristics

**Figure 2:**
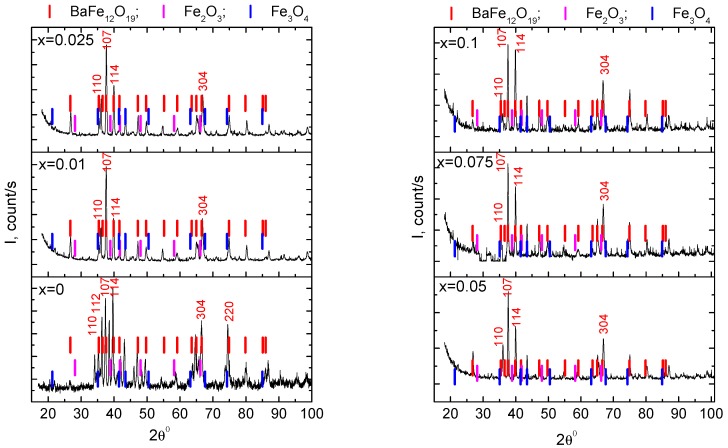
X-ray diffraction patterns of Ba(Fe_1−x_Ga_x_)_12_O_19_ (0 ≤ *x* ≤ 0.1) powders.

Figure 2 shows the XRD X-ray diffraction patterns of initial Ba(Fe_1−x_Ga_x_)_12_O_19_ (0 ≤ *x* ≤ 0.1) powders measured at the room temperature. The calculated positions of reflections for BaFe_12_O_19_ (having a hexagonal lattice P6_3_/mmc with parameters a = 5.887 Å, b = 5.887 Å and c = 23.2 Å and the positions of reflections for iron oxides Fe_2_O_3_ and Fe_3_O_4_ are also marked in the graphs. X-ray diffraction data analysis shows that the barium hexaferrite phase, the unit cell of which is a hexagon and belongs to the space group P6_3_/mmc, prevails in the investigated powders. These data are in accordance with the results obtained previously for similar compounds [15,31,32].

As for the unsubstituted sample of barium hexaferrite (*x* = 0), it is visible from the XRD pattern (Figure 2) that the main characteristic XRD peaks of this sample are located at 2θ = 35.44°, 36.34°, 37.62°, 39.88°, 66.86° and 74.46° and they coincide well with the calculated values which correspond to (110), (112), (107), (114), (304) and (220) reflections of BaFe_12_O_19_, respectively.

Some fairly intense reflections do not correspond to the data calculated for barium hexaferrite. One of the most intense reflections of that sample was observed at 2θ = 38.40° (between (107) and (114) BaFe_12_O_19_ reflections). It is the closest angle to the position calculated for (112) reflection of Fe_2_O_3_, but szs strongly biased in comparison with it. Intense reflection is also observed at 2θ = 43.440, which does not coincide with the available data calculated for BaFe_12_O_19_. Perhaps the reflection at 43.44° is a (222) reflection for Fe_3_O_4_, but this is doubtful, since the most intense line for Fe_3_O_4_ should be (311) at 2θ = 41.46°, and this was not observed for our sample. The reflection with the position of 2θ = 67.59° can be identified as (511) for Fe_3_O_4_, but its intensity is also lower than the expected tabulated values. Therefore, both Fe_3_O_4_ and Fe_2_O_3_ phases in the studied sample, if any, were estimated to have a small amount.

As for the Ga-substituted samples of barium hexaferrite Ba(Fe_1−x_Ga_x_)_12_O_19_ (0.01 ≤ *x* ≤ 0.1), it is clear from the XRD patterns (Figure 2) that the main characteristic XRD peaks of these samples coincide well with the calculated values which correspond to (110), (107), (114) and (304) reflections of BaFe_12_O_19_, respectively. Ga-substituted samples in comparison with unsubstituted BaFe_12_O_19_ differ in the distribution of the main reflection intensities. Namely, the (107) reflection of BaFe_12_O_19_ phase in Ga-substituted samples became the most intense instead of the (114) reflection of unsubstituted BaFe_12_O_19_ (see data in Table 1). In addition, the intensities of (112) and (222) reflections were significantly reduced in Ga-substituted hexaferrites. The unidentified reflection at 2θ = 38.40° of unsubstituted BaFe_12_O_19_ (between (107) and (114) BaFe_12_O_19_ reflections) was not observed for all Ga-substituted samples.

XRD data analysis of the hexaferrite powders showed that the relative intensities of (107) and (114) reflections, which correspond to the inclined *c*-axis orientation, are higher than other peaks. These results indicate that the materials we investigated have a polycrystalline structure with a random grains orientation.

The average crystallite size in Ba(Fe_1−x_Ga_x_)_12_O_19_ (0 ≤ *x* ≤ 0.1) was determined using the well-known Scherrer formula from the line broadening of the diffraction profile of the strongest peaks of (107) and (114) planes:

D = kλ/h_1/2_ × cosθ, (4)

where D is the average crystallite size, k = 0.9—Scherrer constant for spheres, λ is the radiation wavelength (λ_Co_ = 1.790263 Å), h_1/2_ = Full width at half maximum—FWHM (in radians) and θ is the position of the reflection (in radians). The average size of the crystallites in the Ba(Fe_1−x_Ga_x_)_12_O_19_ (0 ≤ *x* ≤ 0.1) powders (using the FWHM of (107) and (114) reflection) was calculated by Equation (4). The values are presented in Table 2.

**Figure 3:**
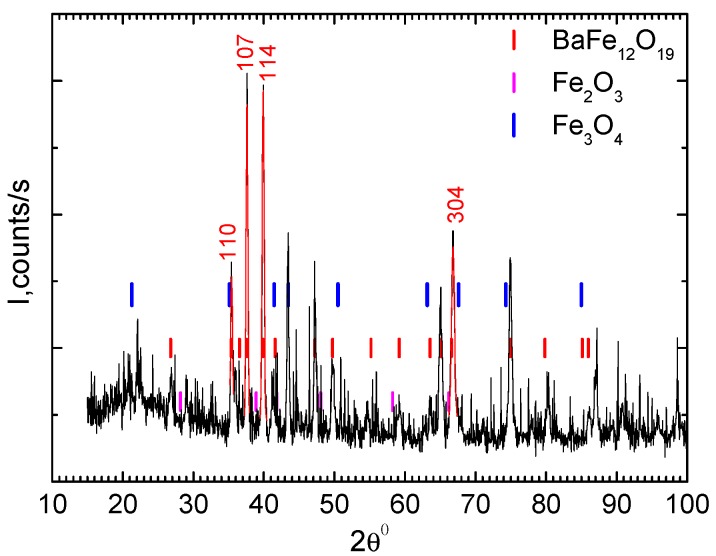
X-ray diffraction patterns of 30 wt.% Ba(Fe_1−x_Ga_x_)_12_O_19_/epoxy (*x* = 0.1) composite.

The average crystallite size of Ba(Fe_1−x_Ga_x_)_12_O_19_ (0 ≤ *x* ≤ 0.1) was estimated to be about 33–37 nm and it depended on the content x of Ga in Ba(Fe_1−x_Ga_x_)_12_O_19_ (0 < *x* < 0.1) (see Table 2). Therefore, the Ba(Fe_1−x_Ga_x_)_12_O_19_ (0 ≤ *x* ≤ 0.1) crystallites were single-domain which correlates well with data for BaM are 460 nm [33]. At average crystal size 10 nm, substituted M-type hexaferrites were in superparamagnetic state [34]. The XRD pattern of 30 wt.% Ba(Fe_1−x_Ga_x_)_12_O_19_ (*x* = 0.1)/epoxy composite is shown in Figure 3. The pattern shows sharp peaks corresponding to the main reflection of Ba(Fe_1−x_Ga_x_)_12_O_19_ (*x* = 0.1) which means that this composite contained crystalline barium hexaferrite. The preponderance of crystalline peaks of BaM was attributed to its encapsulation by epoxy resin. The relative intensity of 30 wt.% Ba(Fe_1−x_Ga_x_)_12_O_19_/epoxy composite is weakened compared to pure BaM powder. Again, using the above Scherrer formula with the line broadening of the diffraction profile of the strongest peaks of the planes (107) and (114), the average crystallite size of 30 wt.% Ba(Fe_1−x_Ga_x_)_12_O_19_/epoxy composite was estimated at about 35–36 nm which is consistent with the data obtained for Ba(Fe_1−x_Ga_x_)_12_O_19_ (0.01 ≤ *x* ≤ 0.1) powders.

**Figure 4:**
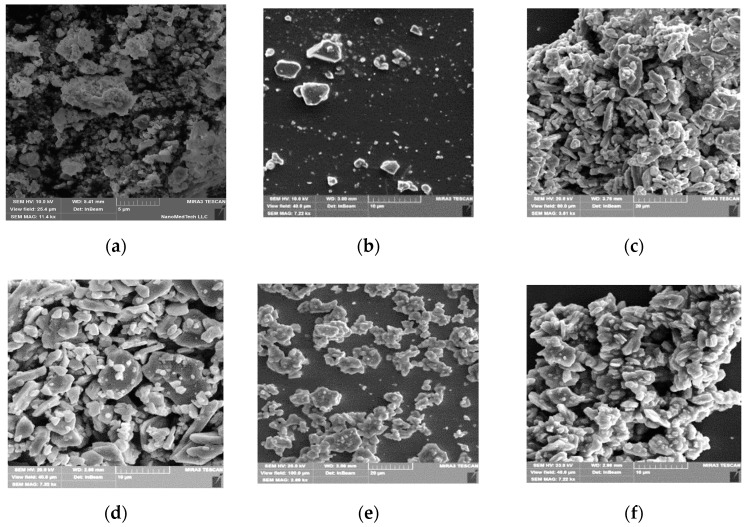
Scanning electron microscope (SEM)-images of the Ba(Fe_1−x_Ga_x_)_12_O_19_ powders: (**a**) *x* = 0; (**b**) *x* = 0.01; (**c**) *x* = 0.025; (**d**) *x* = 0.05; (**e**) *x* = 0.075; (**f**) *x* = 0.1.

Figure 4 demonstrates scanning electron microscopy images of Ba(Fe_1−x_Ga_x_)_12_O_19_ (0.01 ≤ *x* ≤ 0.1) fillers. The samples are densely packed polycrystals (>98%).

The change in grain size is more significant for samples with low Ga concentration. The crystallites combine and form an entire ceramic. A certain dispersion of particle sizes was characteristic for all samples. This is in good agreement with our previous data: the grain size variation interval was between 0.223 and 1.279 μm for *x* = 0.01, and 52.4% of the crystallites were from 0.740 μm to 0.860 μm. Grains with sizes smaller than 0.170 μm or larger than 1.400 μm were not detected. The precise value of the average crystallite size of 〈D〉 ≈ 0.873 μm for *x* = 0.01 was obtained from quantitative stereological analysis. The average crystallite size increased to ≈ 950 nm with an increase in the substitution coefficient to *x* = 0.1 [14].

**Figure 5:**
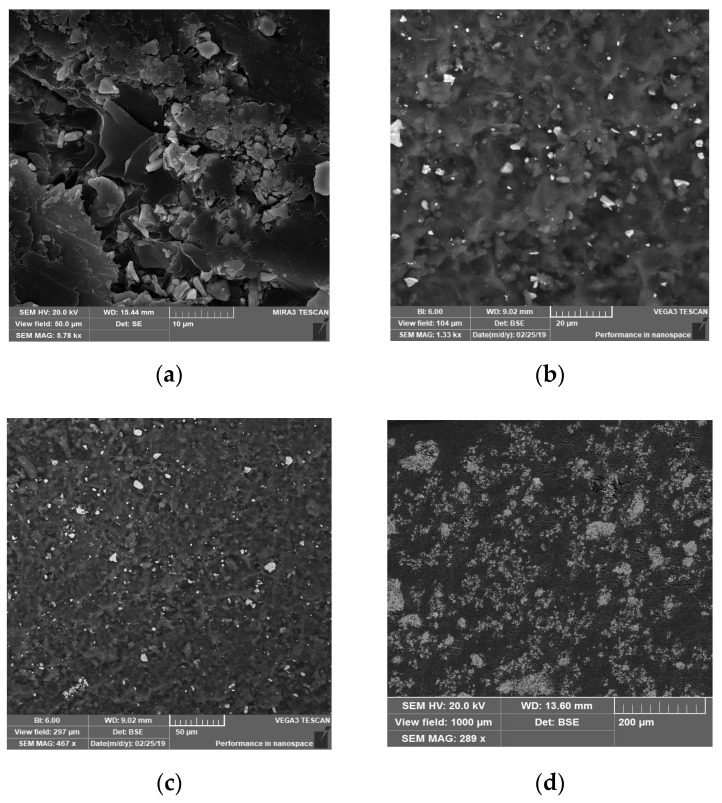
Microstructure of 30 wt.% Ba(Fe_1−x_Ga_x_)_12_O_19_ (*x* = 0.1)/epoxy composite at different magnifications: (**a**) 5780×; (**b**) 1330×; (**c**) 467×; (**d**) 289×.

Figure 5 shows the microstructure of 30 wt.% Ba(Fe_1−x_Ga_x_)_12_O_19_/epoxy (*x* = 0.1) composite at different magnifications. It can be seen that individual particles and aggregates of *x* = 0.1 are coated with epoxy in different positions. As shown in Figure 5a, dispersed individual particles of *x* = 0.1 and their aggregates were found in the epoxy matrix. The aggregates are 10–20 μm in size, but there are many globules with a typical size of about 50 μm. These globules mainly have a regular spherical form. SEM analysis shows a slightly disturbed uniform distribution of ferrite globules in the polymer matrix of 30 wt.% Ba(Fe_1−x_Ga_x_)_12_O_19_ (*x* = 0.1)/epoxy composite samples.

### 3.2. Magnetic Properties

**Figure 6:**
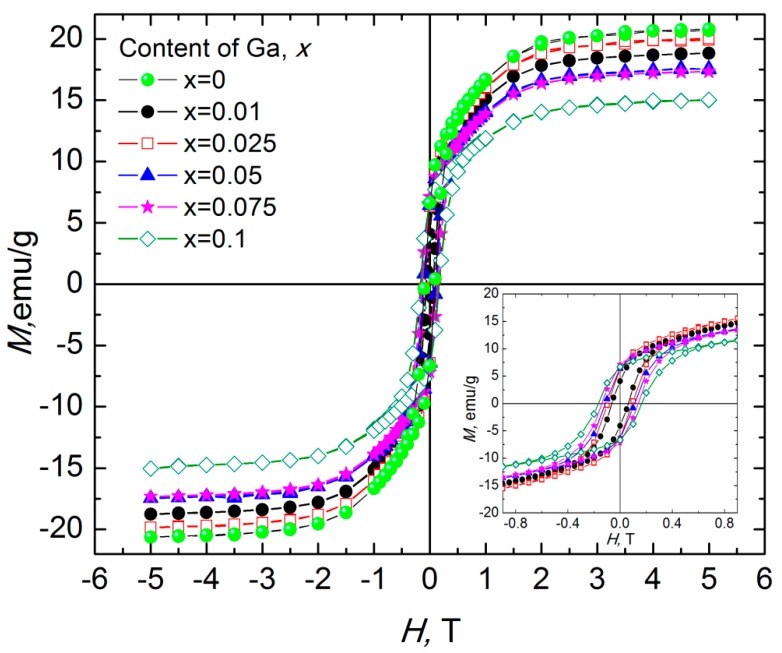
Field dependence of the specific magnetization at room temperature for 30 wt.% Ba(Fe_1−x_Ga_x_)_12_O_19_/epoxy composites (0 ≤ *x* ≤ 0.1).

We discussed in [15,32] the magnetic parameters of substituted with diamagnetic ions polycrystalline samples of Ba(Fe_1−x_Ga_x_)_12_O_19_ (0 ≤ *x* ≤ 0.1). Here, we present the magnetic properties of epoxy-based composites, namely, 30 wt.% Ba(Fe_1−x_Ga_x_)_12_O_19_/epoxy (0 ≤ *x* ≤ 0.1) with random distribution of the filler. Typical M–H field magnetization curves of the composites at room temperature are shown in Figure 6. The values of saturation magnetization (M_S_), residual magnetization (Mr), saturation magnetic field (H_sat_) and coercivity (H_C_) are given in Table 3. Dependencies were measured for two orientations of each sample relative to the direction of the magnetic field H (where S is the designation of the sample orientation).

**Figure 7:**
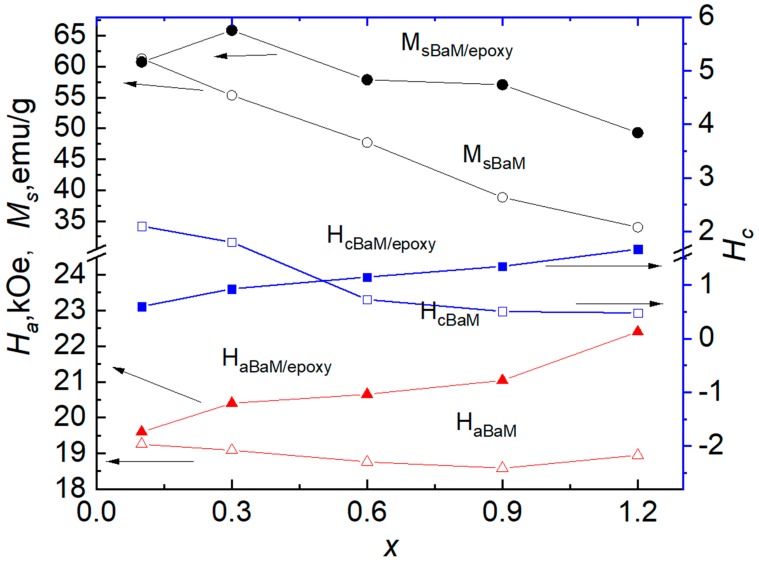
Saturation magnetization (M_s_), coercivity (H_C_) and anisotropy field (H_a_) of Ba(Fe_1−x_Ga_x_)_12_O_19_ (0 ≤ *x* ≤ 0.1) powders and of 30 wt.% Ba(Fe_1−x_Ga_x_)_12_O_19_/epoxy composites with 0 ≤ *x* ≤ 0.1 versus Ga concentration (*x*).

The magnetization field dependence for 30 wt.% Ba(Fe_1−x_Ga_x_)_12_O_19_/epoxy composites shows clear hysteresis behavior. Such magnetization field dependence is a characteristic for all investigated 30 wt.% Ba(Fe_1−x_Ga_x_)_12_O_19_/epoxy composites as a consequence of magnetic response of the Ba(Fe_1−x_Ga_x_)_12_O_19_ component. It is assumed that the total magnetization of the composite is formed only due to Ba(Fe_1−x_Ga_x_)_12_O_19_ filler, as epoxy is non-magnetic. As can be seen from the data in Figure 6 and Table 3, the coercive force H_C_ increases monotonically from 0.060 to 0.166 T with an increase of Ga content in the filler while the saturation magnetization M_S_ decreases with Ga content increase (Figure 7). Nevertheless, an insignificant maximum of M_S_ was observed for 30 wt.% Ba(Fe_1−x_Ga_x_)_12_O_19_/epoxy composite. One can see that M_S_ changes from 20.31 to 14.70 emu/g for composites with different contents of Ga (0 ≤ *x* ≤ 0.1) in the filler. The values of H_C_, M_r_ and M_S_ almost coincide for the magnetic moment measured in both orientations of 30 wt.% Ba(Fe_1−x_Ga_x_)_12_O_19_/epoxy composites samples. It is assumed that small deviations of the measured magnetic moment in this case indicate heterogeneity of the filler distribution in the epoxy matrix. The dependencies of H_a_ (*x*), H_C_ (*x*) and M_S_ (*x*) of Ba(Fe_1−x_Ga_x_)_12_O_19_ (0 ≤ *x* ≤ 0.1) powders and 30 wt.% Ba(Fe_1−x_Ga_x_)_12_O_19_/epoxy composites (0 ≤ *x* ≤ 0.1) are presented for comparison in Figure 7.

As shown in [35], the specific saturation magnetization M_rfc_ of rubber–ferrite composites was found to be linearly dependent on the mass fraction of ferrite and obeys the following general relation:

M_rfc_ = M_S_ W_f_, (5)

where M_S_ and W_f_ are the saturation magnetization and weight fraction of the filler, respectively.

As seen in Figure 7, the saturation magnetization M_S_ (*x*) decreases with increasing Ga content x in the filler for both materials investigated, but the value of M_S_ is higher for Ba(Fe_1−x_Ga_x_)_12_O_19_/epoxy (0 ≤ *x* ≤ 0.1) composites. As it was shown in our previous paper [15], such behavior of the specific saturation magnetization for the Ba(Fe_1−x_Ga_x_)_12_O_19_ (0 ≤ *x* ≤ 0.1) polycrystalline samples indicates a decrease in the maximum magnetic energy with an increase in the concentration of Ga^3+^ cations and the absence of abrupt anomalies. It means a decrease in deviations from the linear dependence of magnetic energy with increasing substitution ratio, which testifies to the hypothesis of a statistical distribution of Ga^3+^ cations between different nonequivalent crystallographic positions in the M-type barium hexaferrite structure. The polymer coating on magnetic particles obviously affects the contributions of the surface anisotropy, shape anisotropy and interface anisotropy to the total anisotropy [36,37]. The magnetic parameters of Ba(Fe_1−x_Ga_x_)_12_O_19_/epoxy composites are higher than the corresponding parameters of pure Ba(Fe_1−x_Ga_x_)_12_O_19_ (0 ≤ *x* ≤ 0.1) polycrystalline samples, while the shape of M_S_ (x) dependencies remains unchanged. Figure 7 shows that coercivity H_C_ of Ba(Fe_1−x_Ga_x_)_12_O_19_ polycrystals with low content of Ga (*x* = 0.01) notably decreases from 2.1 kOe to 0.6 kOe when they are embedded into the polymer matrix of the composite. A monotonic increase in H_C_ is observed with increasing content of Ga in Ba(Fe_1−x_Ga_x_)_12_O_19_/epoxy composites.

The magnetization-field dependence for 30 wt.% Ba(Fe_1−x_Ga_x_)_12_O_19_/epoxy composites is relatively small, which may be due to the prevalence of domain rotation in the high-field region. The relationship between M and H in this region is called the “law of approach to saturation” and is usually written as [38]:

M = M_S_ [1 − (A/H) − (B/H^2^)] − ΘH, (6)

where M_S_ is the saturation magnetization of the domains. The term ΘH represents the field-induced increase in the saturation magnetization of the domains, or forced magnetization; this term is usually small at temperatures well below the Curie point and may often be neglected. Constant A is generally interpreted as a result of inclusions and/or microstress, and B is due to magnetocrystalline anisotropy. The magnetization data in a field range of about 3 kOe were plotted against 1/H^2^ in [17], and straight lines were obtained, indicating that both A and ΘH are negligible in the aforementioned magnetic field range.

Saturation magnetization values for the samples were obtained from the intersections of the straight lines. The slopes of the lines were used to determine the anisotropy field H_a_ from the relationships [17]:

B = H_a_^2^/15. (7)

The first anisotropy constant was evaluated using the relation [38]:

K_1_ = (H_a_ M_S_)/2. (8)

**Figure 8:**
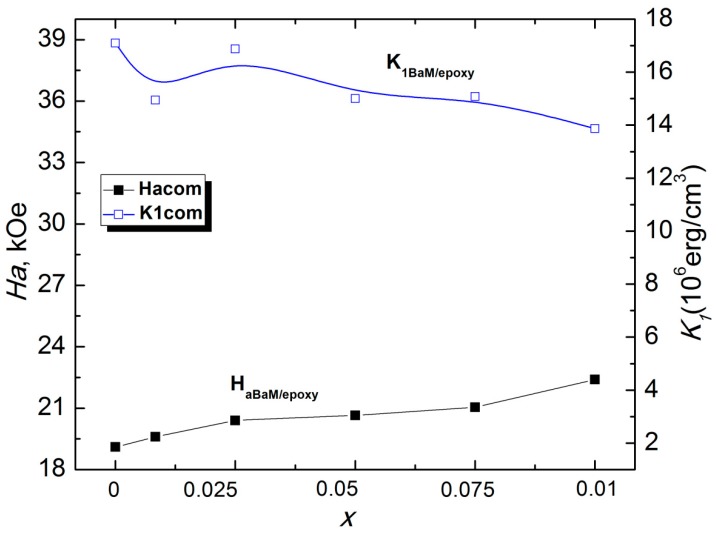
Anisotropy field H_a_ and the first anisotropy constant K_1_ of 30 wt.% Ba(Fe_1−x_Ga_x_)_12_O_19_/epoxy composites versus Ga concentration (0 ≤ *x* ≤ 0.1).

The anisotropy field H_a_ and anisotropy constant K_1_ of 30 wt.% Ba(Fe_1−x_Ga_x_)_12_O_19_/epoxy composites (0 ≤ *x* ≤ 0.1) were calculated using Equations (7) and (8), and the results are presented in Figure 8.

### 3.3. Microwave Properties

**Figure 9:**
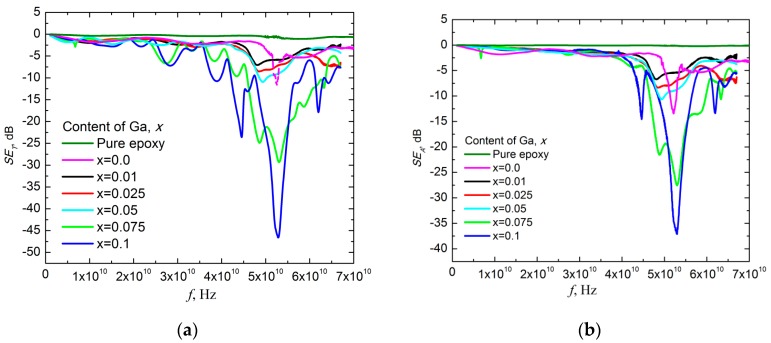
Millimeter wave transmittance spectra (**a**) and absorption spectra (**b**) of all 30 wt.% Ba(Fe_1−x_Ga_x_)_12_O_19_/epoxy composites (0 ≤ *x* ≤ 0.1) and pure epoxy.

Transmittance spectra of 30 wt.% Ba(Fe_1−x_Ga_x_)_12_O_19_/epoxy composites (0 ≤ *x* ≤ 0.1) were recorded in millimeter wave range and are shown in Figure 9a. As seen in Figure 9a, the values of shielding efficiency (SE_T_) are quite small up to frequencies of 40–45 GHz.

It is known [39] that the value of total material shielding efficiency SE_T_ is equal to the sum of the absorption coefficient SE_A_, reflection coefficient SE_R_ and correction factor SE_I_, which takes into account multiple reflections in thin high-conductive shields or in shields with a small absorption coefficient:

SE_T_ = SE_A_ + SE_R_ + SE_I_. (9)

SE_I_ is negligible in cases when SE_A_ exceeds 10 dB.

**Figure 10:**
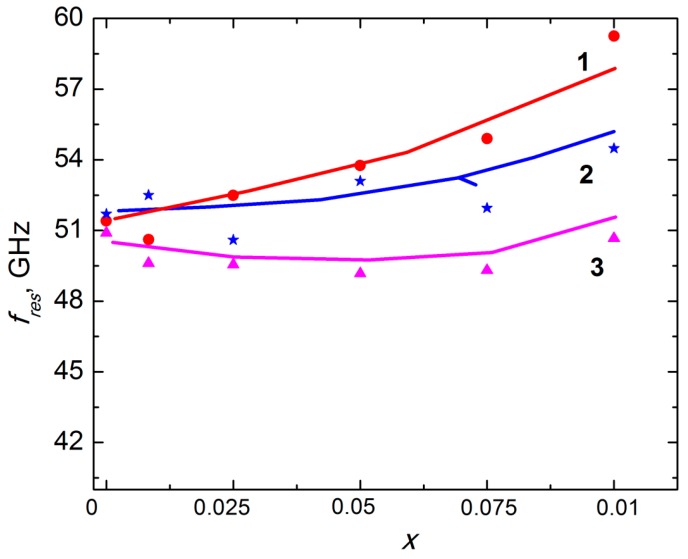
Resonance frequency f_res_ of the samples versus Ga concentration. *x*: 1—f_res_ for 30 wt.% Ba(Fe_1−x_Ga_x_)_12_O_19_/epoxy composites (0 ≤ *x* ≤ 0.1), calculated using the DC direct current magnetization results; 2—f_res_ for 30 wt.% Ba(Fe_1−x_Ga_x_)_12_O_19_/epoxy composites (0 ≤ *x* ≤ 0.1) determined by microwave measurements; 3—f_res_ [32] for Ba(Fe_1−x_Ga_x_)_12_O_19_ (0 ≤ *x* ≤ 0.1) polycrystalline solid solutions.

As seen in Figure 9a,b the main contribution to the total material shielding efficiency SE_T_ of 30 wt.% Ba(Fe_1−x_Ga_x_)_12_O_19_/epoxy composites (0 ≤ *x* ≤ 0.1) was the absorption of electromagnetic waves in the materials. The most intensive EMR absorption was observed in 40–50 GHz frequency range for all the above-mentioned composite samples. The NFMR in barium hexaferrite powders provides high EMR absorption in the indicated frequency range. The transmission value at the global minimum of microwave transmission spectrum determines the resonant transmission A_res_. The frequency in the global minimum of microwave transmission spectrum determines the resonant transmission frequency f_res_. The global minimum width, measured at A_res_/2, i.e., half of the resonant transmission value, determines the width of the absorption band W_res_—the bandwidth. One can see in Figure 9a,b that all three above-mentioned quantities are sensitive to the substitution ratio *x*. The NFMR frequency f_res_ was measured at the half of the bandwidth W_res_/2 and the results are presented in Figure 10. It was assumed that the determined frequency was associated with f_res_ in Ba(Fe_1−x_Ga_x_)_12_O_19_ ferrite filler. f_res_ in unsubstituted BaFe_12_O_19_ is near 50 GHz and can be calculated as [17]:

f_res_ = γ(H_a_ − 4π M_S_), (10)

if the demagnetizing effects are neglected.

Here γ is the gyromagnetic ratio. Using the above-stated experimental results on the anisotropy field and saturation magnetization, the NFMR frequency f_res_ for 30 wt.% Ba(Fe_1−x_Ga_x_)_12_O_19_/epoxy composites (0 ≤ *x* ≤ 0.1) was evaluated and the values are also presented in Figure 10. As seen in Figure 10, f_res_ values determined from DC magnetization measurements are in a rather good agreement with f_res_ values determined by microwave measurements.

As it was shown in our previous publications [31,32], the peak of absorption is shifted towards higher frequencies with increasing x in Ba(Fe_1−x_Ga_x_)_12_O_19_ (0 ≤ *x* ≤ 0.1) powders (see Figure 10). However, the concentration dependence of the resonant frequency for Ba(Fe_1−x_Ga_x_)_12_O_19_ (0 ≤ *x* ≤ 0.1) powders is non-monotonic and demonstrates the minimum at *x* = 0.05. As one can see, encapsulation of Ba(Fe_1−x_Ga_x_)_12_O_19_ particles in epoxy leads to the resonance frequency increase for 30 wt.% Ba(Fe_1−x_Ga_x_)_12_O_19_/epoxy composites in comparison with pure Ba(Fe_1−x_Ga_x_)_12_O_19_ (0 ≤ *x* ≤ 0.1) powders and it essentially changes the shape of f_res_(*x*) dependencies.

Higher f_res_ values in 30 wt.% Ba(Fe_1−x_Ga_x_)_12_O_19_/epoxy composites (0.01 ≤ *x* ≤ 0.1) in comparison with f_res_ in Ba(Fe_1−x_Ga_x_)_12_O_19_ (0 ≤ *x* ≤ 0.1) powders could be explained using the results of magnetic measurements.

As our previous research on BaFe_12_O_19_/epoxy polymer composite magnetic properties has shown [24], encapsulation of the magnetic powder in a polymer core leads to a change in chemical bonds on the surface of the particles. This causes a decrease in the saturation magnetization of these magnetic particles and affects the contributions of the surface anisotropy, the shape anisotropy and the interface anisotropy to the net anisotropy. So, polymer coating of fine particles and subsequent changes of their magnetic characteristics (in particular, a decrease in the saturation magnetization) in a polymer composite produces a shift of f_res_ towards higher frequencies.

It is known that the magnitude of the absorption coefficient SE_A_ is in direct proportion to the sample thickness t and can be expressed by the following equation [40]:

SE_A_ = −8.7 t × (σ_T_ × πfµ)^1/2^, (11)

where σ_T_ is total electrical conductivity which is composed of frequency dependent and independent components; µ is the permeability.

**Figure 11:**
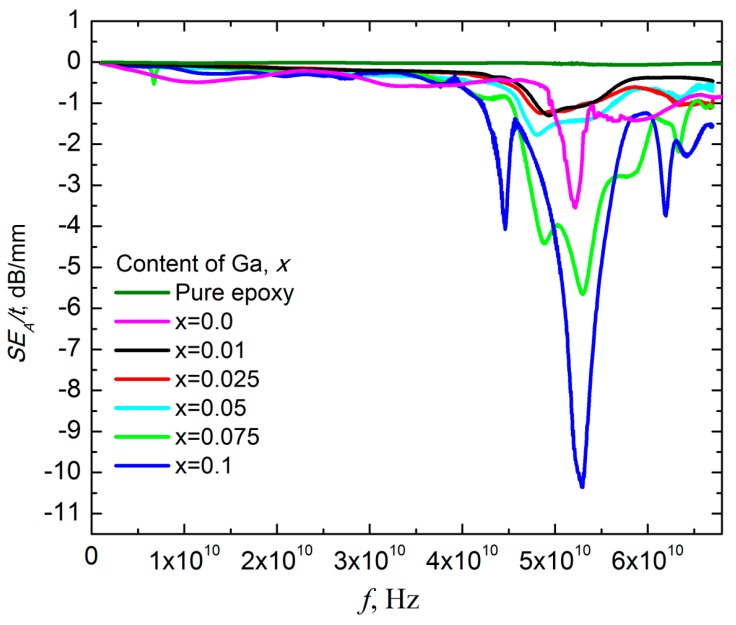
The adjusted absorption spectra SE_A_/t for 30 wt.% Ba(Fe_1−x_Ga_x_)_12_O_19_/epoxy composites (0 ≤ *x* ≤ 0.1) and pure epoxy.

So, in order to evaluate the influence of the Ga content in hexaferrite fillers on the absorption spectra of 30 wt.% Ba(Fe_1−x_Ga_x_)_12_O_19_/epoxy composites (0 ≤ *x* ≤ 0.1), the adjusted value of the absorption coefficient SE_A_/t was introduced, and the frequency dependencies of SE_A_/t are presented in Figure 11.

**Figure 12:**
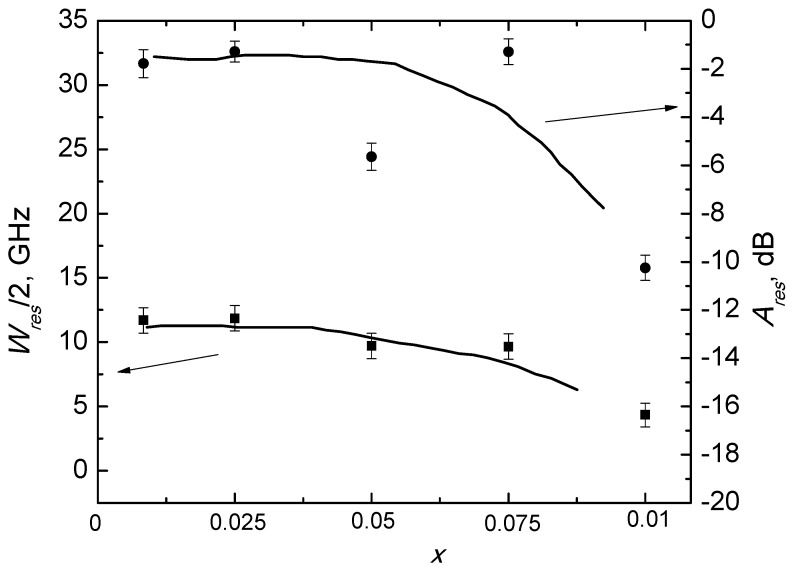
Resonant absorption value and absorption band versus Ga content in Ba(Fe_1−x_Ga_x_)_12_O_19_ filler.

These data were used for A_res_ and W_res_ dependencies on the Ga content calculated for Ba(Fe_1−x_Ga_x_)_12_O_19_. These dependencies are presented in Figure 12.

As shown in Figure 11 and Figure 12, W_res_ decreases monotonically with an increase in the substituent concentration of Ga^3+^. It is obvious that peculiar properties of Ba(Fe_1−x_Ga_x_)_12_O_19_ determined the frequency dependence of the EMR absorption process in the Ba(Fe_1−x_Ga_x_)_12_O_19_/epoxy composites.

In polycrystalline ferrites, the total NFMR linewidth △H depends crucially on the superposition of intrinsic and extrinsic contributions [41]:

△H = △H_i_ + △H_a_ + △H_p_, (12)

where c is the intrinsic linewidth, △H_a_ is the crystalline anisotropy contribution and△H_p_ is the porosity induced line broadening contribution. Karim et al. in [42] speculated that barium hexaferrites have an intrinsic linewidth of 0.3–0.4 Oe/GHz. Parameter △Ha~0.7 H_a_ and relates to the crystalline anisotropy. Parameter △Hp1.5(4πM_S_)P accounts for porosity (P) induced linewidth broadening contributions [43,44].

The main role in W_res_ broadening in pure Ba(Fe_1−x_Ga_x_)_12_O_19_ powders with different contents of Ga is played by static inhomogeneities, such as impurity cations, which lead to increases in the magnetocrystalline anisotropy; and W_res_ values increase monotonically, increasing the substitution ratios. As the samples were obtained at the same time and using identical technology, they had identical morphologies in terms of their crystallites. However, it can be supposed that P does not appreciably differ in the samples with different x and the changes of P are negligible. A_res_ and W_res_ increases monotonically with an increase in x in the 30 wt.% Ba(Fe_1−x_Ga_x_)_12_O_19_/epoxy composites. However, the values of ΔH were greater than those reported for BaFe_12−x_Ga_x_O_19_, which can be explained by a random distribution of the anisotropy axes in the crystallites and by an increase of porosity and the random orientations of crystallites themselves [45,46,47,48,49]. This cause of A_res_ variation from point to point within the material, in turn, broadening the resonance line.

## 4. Conclusions

Ga-substituted barium hexaferrite powders were synthesized via two-step solid state reactions and characterized by XRD and SEM methods. Calculations of the crystallite size of Ba(Fe_1−x_Ga_x_)_12_O_19_ (0.01 ≤ *x* ≤ 0.1) grains using the Scherrer formula showed that the Ba(Fe_1−x_Ga_x_)_12_O_19_ crystallites we investigated (not grains) are single domain, and the crystallite size remains unchanged in composites with Ba(Fe_1−x_Ga_x_)_12_O_19_ filler. Encapsulation of Ga-substituted barium hexaferrite particles in the epoxy matrix allowed us to prepare microwave absorbing composites. The magnetization-field dependence measurements showed that coercive force H_C_ of Ba(Fe_1−x_Ga_x_)_12_O_19_/epoxy composites increases monotonically from 0.060 to 0.166 T, while the saturation magnetization M_S_ decreases with increasing Ga content in filler. The values of magnetic parameters of Ba(Fe_1−x_Ga_x_)_12_O_19_/epoxy composites were higher than that of pure Ba(Fe_1−x_Ga_x_)_12_O_19_ polycrystalline samples, while the shape of M_S_ (*x*) dependencies remained unchanged. Calculation of anisotropy field H_a_ and anisotropy constant K_1_ for 30 wt.% Ba(Fe_1−x_Ga_x_)_12_O_19_/epoxy composites was carried out. Studies of Ba(Fe_1−x_Ga_x_)_12_O_19/_epoxy composites with different substitution ratio of gallium filler (0.01 ≤ *x* ≤ 0.1) showed a noticeable effect of Ga content on the microwave characteristics. The most intensive EMR absorption was observed in the 49–54 GHz frequency range for all composites tested, which was attributed to the effect of NFMR in Ba(Fe_1−x_Ga_x_)_12_O_19_ hexaferrites. Higher resonance frequencies of 30 wt.% Ba(Fe_1−x_Ga_x_)_12_O_19_/epoxy composites (0.01 ≤ *x* ≤ 0.1) in comparison with resonance frequencies of Ba(Fe_1−x_Ga_x_)_12_O_19_ (0.01 ≤ *x* ≤ 0.1) powders could be explained by a decrease in the saturation magnetization of the magnetic particles due to their encapsulation in the epoxy which affects the contributions of the surface anisotropy, the shape anisotropy and the interface anisotropy to the net anisotropy. The absorption band W_res_ decreases monotonically with an increase in Ga concentration in the hexaferrite filler while the resonant amplitude A_res_ increases.

## Figures and Tables

**Table 1:** Comparison of the relative reflection intensities in the XRD X-ray diffraction patterns for Ba(Fe_1−x_Ga_x_)_12_O_19_ (0.01 ≤ *x* ≤ 0.1) powders.

The Content of Ga in Ba(Fe_1−x_Ga_x_)_12_O_19_, *x*	Reflection Order	Reflection Position, 2θ, Deg	Full Width at Half Maximum (FWHM), Δ2θ, Deg	I/I_107_ Relation
0	110	35.27	0.282	0.46
	107	37.40	0.313	1
	114	39.65	0.336	1.14
	304	66.53	0.625	0.84
0.01	110	35.44	0.247	0.12
	107	37.62	0.344	1
	114	39.88	0.355	0.44
	304	66.86	0.393	0.35
0.025	110	35.48	0.242	0.12
	107	37.68	0.34	1
	114	39.95	0.33	0.55
	304	66.91	0.472	0.39
0.05	110	35.51	0.248	0.15
	107	37.69	0.326	1
	114	39.98	0.328	0.62
	304	66.91	0.45	0.40
0.075	110	35.50	0.336	0.37
	107	37.65	0.313	1
	114	39.94	0.318	0.78
	304	66.87	0.444	0.50
0.1	110	35.45	0.346	0.42
	107	37.61	0.34	1
	114	39.90	0.34	1
	304	66.82	0.53	0.57

**Table 2:** The average crystallite size in Ba(Fe_1−x_Ga_x_)_12_O_19_ (0 ≤ *x* ≤ 0.1), determined by the Scherrer formula.

The Content of Ga in Ba(Fe_1−x_Ga_x_)_12_O_19_, *x*	The Average Size of the Crystallites, nm (Calculation by (107) Reflection)	The Average Size of the Crystallites, nm (Calculation by (114) Reflection)
0	37.1	35.6
0.01	33.8	33.9
0.025	34.3	36.5
0.05	35.7	36.7
0.075	37.2	37.8
0.1	34.2	35.4

**Table 3:** Comparison of the coercivity (H_C_), residual magnetization (M_r_), saturation magnetization (M_S_) and saturation magnetic field (H_sat_) for 30 wt.% Ba(Fe_1−x_Ga_x_)_12_O_19_/epoxy composites with 0 ≤ *x* ≤ 0.1.

*x* of Ga in Ba(Fe_1−x_Ga_x_)_12_O_19_	Hc (kOe)		M_S_ (emu/g)		Mr(emu/g)		Mr/M_S_		H_sat_ (T)	
	S∥B	S⊥B	S∥B	S⊥B	S∥B	S⊥B	S∥B	S⊥B	S∥B	S⊥B
0	0.97	0.97	20.31	20.07	6.92	6.74	0.34	0.33	3.17	3.28
0.01	0.60	0.64	18.84	18.26	4.08	4.26	0.22	0.23	3.32	2.82
0.025	0.93	0.93	19.96	19.90	6.36	6.20	0.32	0.31	3.18	3.37
0.05	1.08	1.15	17.53	17.48	6.43	6.34	0.37	0.36	2.95	3.23
0.075	1.37	1.35	17.29	17.11	7.13	6.91	0.41	0.40	2.79	3.21
0.1	1.66	1.67	14.94	14.70	6.67	6.50	0.45	0.44	3.16	2.79
